# Video Process Mining and Model Matching for Intelligent Development: Conformance Checking

**DOI:** 10.3390/s23083812

**Published:** 2023-04-07

**Authors:** Shuang Chen, Minghao Zou, Rui Cao, Ziqi Zhao, Qingtian Zeng

**Affiliations:** School of Computer Science and Engineering, Shandong University of Science and Technology, Qingdao 266000, China

**Keywords:** video processing, process model, Petri net, conformance checking

## Abstract

Traditional business process-extraction models mainly rely on structured data such as logs, which are difficult to apply to unstructured data such as images and videos, making it impossible to perform process extractions in many data scenarios. Moreover, the generated process model lacks analysis consistency of the process model, resulting in a single understanding of the process model. To solve these two problems, a method of extracting process models from videos and analyzing the consistency of process models is proposed. Video data are widely used to capture the actual performance of business operations and are key sources of business data. Video data preprocessing, action placement and recognition, predetermined models, and conformance verification are all included in a method for extracting a process model from videos and analyzing the consistency between the process model and the predefined model. Finally, the similarity was calculated using graph edit distances and adjacency relationships (*GED_NAR*). The experimental results showed that the process model mined from the video was better in line with how the business was actually carried out than the process model derived from the noisy process logs.

## 1. Introduction

Business processes store information about the execution of information systems in event logs, and process mining extracts useful information from the event logs [1]. Process mining’s purpose is to compare the event logs or the resulting process model in the discovery task with the existing reference model of the same process and to detect issues in the executed process for improvement [2]. The six degrees of parallel platform is a widely used parallel mechanism that can achieve six DOF of motion of a moving platform in space [3]. This study aims to enhance the security of people’s health, improve the medical level further, and increase the confidentiality of people’s private information [4]. Traditional process mining techniques are based on process logs. However, logs cannot be obtained effectively in some scenarios so it is impossible to mine process models that conform to actual scenarios. In addition, some process logs may contain some erroneous or incomplete behaviors [5], which cause a certain deviation between the model obtained by the process mining algorithm and the ideal model. A method of extracting the process models from videos and analyzing the consistency of the process models is proposed.

Due to various problems in the process logs and a large amount of video data emerging [6], it is very necessary to apply process mining to video data. In the era of big data, the Internet is enmeshed in people’s lives and brings conveniences to their production and lives [7]. Most enterprises have complete video data, which can completely record the actual execution of the business process and contains rich business information. The conformance checking of procedural video is based on video data as the source data, and the process model excavated and the process model obtained from the process log containing noise are checked for consistency with the predefined models so that enterprises can improve their production scheduling and achieve a high-level management of business processes [8]. In addition, it can optimize resources, reduce the operating costs of enterprises, and make enterprises obtain greater profits. Compared with process mining using the process logs as data sources, the work of extracting the processes from videos is less. One of the main reasons is the complexity of video processing technology. The continuous development of moving target detection methods such as the self-supervised video [9], the trapezoid normalized pixel [10], the cycleGan [11], and multi-agent systems [12] provide technical support for video moving target detection. A video is a combination of continuous images. Mining potential task sequences in videos face various problems, for example, most videos suffer from frequent changes in lighting, occlusion between objects, etc. The existing video anomaly detection methods can only detect the anomaly of a single behavioral event but do not consider whether the actual execution of the business process in the video conforms to the actual situation. Traditional methods usually rely on good initialization and find difficulty in handling large interframe transformations due to fast camera motion [13]. Yet, there is no framework in place to evaluate the current state of the deployment of digital education at the beginning of the educational process, orient public education policies, and establish a baseline for comparison and future development among developing countries, ensuring that their future workforce is ready for the challenges of the twenty-first century. To this end, a procedural video conformance-checking method based on process mining and model matching is proposed.

The organization of this paper is as follows: Section 2 introduces the related work on process mining; Section 3 gives the framework of the paper and the introduction of key algorithms for each functional module; Section 4 is the specific experiments and analysis; and Section 5 summarizes the work of this paper.

## 2. Related Work

In recent years, process mining has gained extensive attention and accumulated more and more research results. A process mining anonymization algorithm based on genetic algorithms was proposed in [14], which reduces the privacy protection and utility tradeoff problem of process mining to the optimization problem of the activity inhibition set. Synthesizing photo-realistic images from text descriptions is a challenging problem in computer vision and has many practical applications [15]. In [16] they gave a technique to repair the missing events in a log. They used the knowledge gathered from the process model and provided a technique to repair the missing event logs in a trace. This technique gives us an incomplete log analysis. By using the stochastic Petri net model and alignment, we repair the event logs and convert them into Bayesian analysis. An efficient algorithm for mining the nearest neighbor relationships of relational event logs was proposed in [17]. The algorithm used two-level pointers to scan the log data, which has a linear time complexity for the log size, and the demand for memory space was independent of the log size. This algorithm improved the process mining efficiency of relational event logs.

A model mining method of cross-organizational emergency response process (CERP) was proposed in [18]. This method first extended the classical Petri nets (RMPNs) with resource and message attributes, and found the emergency response process models (IERP) within the organization were represented as RMPNs from the emergency exercise process logs. Secondly, it defined the cooperation mode between emergency organizations. Finally, the CERP model was obtained by combining the IERP model with the collaboration model. Text mining techniques were employed to mine the sentiments in different interactions, and then epistemic network analysis (ENA) was used to uncover sentiment changes in the five learning stages of blended learning [19]. In [20], they formalized the problem of the directly-follows graph (DFG) simplification as an optimization, specifically, the problem of finding a sound-spanning subgraph of a DFG with a minimal number of edges and a maximal sum of edge frequencies. Finally, they reported on an evaluation of the efficiency and optimality of the proposed heuristics using 13 real-life event logs.

To sum up, these traditional process discovery methods used process logs as input. However, process logs cannot be obtained effectively in some scenes, which lead to the process extraction work facing huge challenges. Relatively speaking, unstructured video data are more flexible, records the real situation of the business process, and objectively reflects the actual execution process of the process.

## 3. Framework

**Figure 1:**
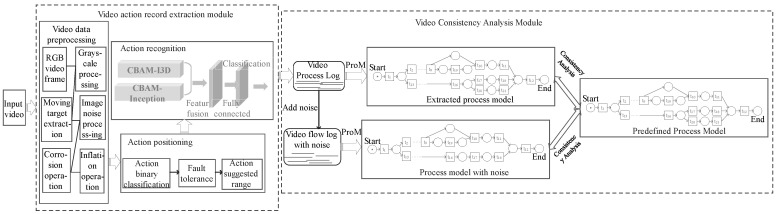
The framework of our approach.

Based on the above analysis, a method for extracting process models from videos and performing conformance checking is proposed, which solves the problems where the process logs cannot be obtained effectively and the process logs are noisy. The framework of our work is shown in Figure 1, including the video action record extraction module and the video conformance checking module. The video action record extraction module first preprocesses the video data, removes image noise and extracts moving targets, and then inputs the preprocessed images into the action localization network to generate action suggestions of different length intervals, and finally identifies the action category label of the action suggestion interval through the action recognition network, and saves the action category label in the log, builds a video flow log, adds some noise on this basis, such as wrong or incomplete behavior, and builds a video flow log with the noise; the purpose of adding noise is to simulate actual process logs with noise in the information system. The video conformance checking module includes generating predefined models and conformance checking. The predefined module defines the standard Petri net process models, and the conformance checking module uses the graph edit distance and the adjacency relationship (*GED_NAR*) method to judge the matching degree of the process model containing noise and the extracted process model with the predefined model.

### 3.1. Video Action Record Extraction Module

The action record extraction of the video includes two parts: video data preprocessing, and action localization and recognition. The goal is to extract process information from the process video. Video data preprocessing can maximize data simplification and reduce computational interference. Motion localization and recognition is the process of filtering and identifying preprocessed images from the video data to complete the process extraction of procedural video.

#### 3.1.1. Video Data Preprocessing

Four techniques are used for the video preprocessing: image grayscale processing, knn-based moving target extraction, image noise processing, and open operation.

(1) Image grayscale processing: The formula is shown in (1), where H and W represent the height of the original video frame and width, *i* ∈ [1*…H*], *j* ∈ [1*…W*].

(1) Gray=max⁡{R(i,j),G(i,j),B(i,j)}

Image grayscale can turn three channels into a single channel, reduce memory space, improve operation speed, increase visual contrast, and highlight the target area.

(2) Moving target extraction: The algorithm’s purpose is to extract the motion information contained in the action in the video. The image is grayed out and still contains useless background information. In order to accurately extract the moving targets, the moving target is extracted by the knn algorithm [21]. Basically, the knn algorithm can accurately extract moving targets, but it is still disturbed by noise.
(3) Image noise processing: The method of median filtering is used to perform nonlinear filtering on grayscale images, so that the target pixel is closer to the real value, thereby eliminating isolated noise points. The calculation method is shown in Formula (2), where *g (x, y)* is the processed image, *f (x, y)* is the original image, *W* is an N*N two-dimensional template, N is usually a positive odd number, and Med represents sorting the gray values in the domain window and taking out the middle value:

(2) g(x,y)=Med{f(x−k,y−l),(k,l∈W)}

(4) Open operation processing: Assuming that *Z* is the target image and *W* is a structural element, the mathematical formula for the opening operation processing of the structural element *W* by the target image *Z* is:

(3) Z∘W=(ZΘW)⨁W

(4) $$Z\Theta W=\left\{x,y|{\left(W\right)}_{xy}\subseteq Y\right\}$$

(5) $$Z⨁W=\{x,y|(W{)}_{xy}\cap X\neq \emptyset \}$$

where *(W)_xy_* represents the translation of the origin of the structuring element *W* to the position of the image pixel (*x*, *y*), the erosion operation is represented by Θ, and the dilation operation is represented by ⨁. The open operations smooth the contours of the moving targets and break up the narrow connection areas and remove the small protrusions from targets.

**Figure 2:**
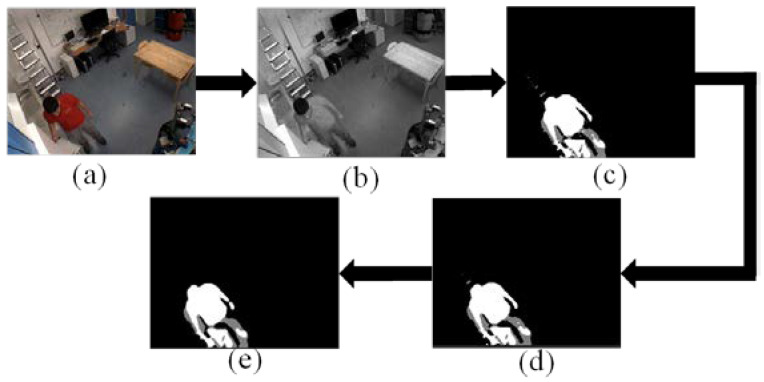
Sample Image of video data preprocessing (**a**) Video frame (**b**) Grayscale image processing image (**c**) Target extraction processing image (**d**) Median filter processing image (**e**) Open operation processing image.

The video data preprocessing can extract the interest points of the actual actions and high-light the potential task sequences in the video. The processing results are shown in Figure 2.

#### 3.1.2. Action Location and Recognition

**Figure 3:**
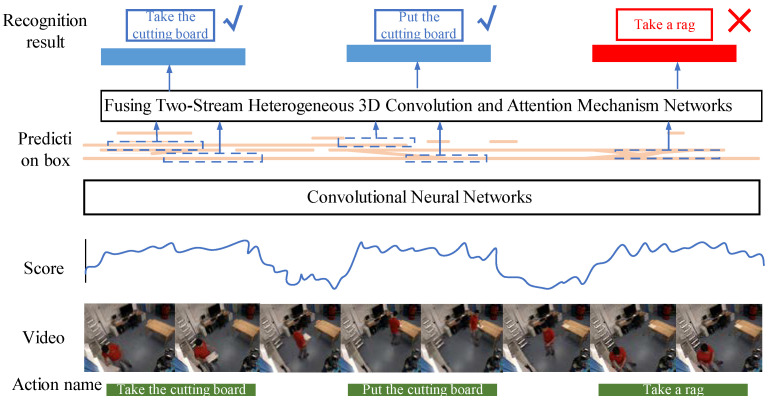
Action location and recognition.

After video data preprocessing, the next step is to locate and recognize actions in the preprocessed image sequence, as shown in Figure 3. For videos, both the target action and the duration of the action are varied. The coherence between actions makes it difficult to locate the start and end points of the action. A binary classification method based on a convolutional neural network is used to distinguish between action and background. To generate the temporal region proposals, our basic idea is to group consecutive snippets with high actionness scores. In a given video, we first sample a sequence of snippets, then use this network to produce actionness scores for them, and finally group them into temporal regions of various granularities. A fault-tolerant processing scheme with high robustness is designed, which can generate action suggestion intervals of different lengths, allowing accurate identification of the duration of the action in the video. With a set of proposed temporal regions, the next stage is to classify them into action classes.

**Figure 4:**
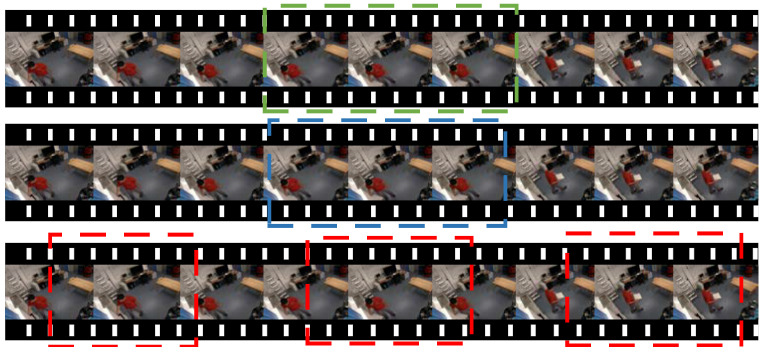
Action instance. The green box represents ground truths, the blue box represents a good prediction, and the red box represents a poor prediction.

In short, we hope that the action proposed interval can cover all kinds of action time. A binary classification network is designed to distinguish action from the background, filtering out which clips contain action and which clips are backgrounds. Firstly, input the preprocessed image into the binary classification network, and each image gets a binary classification result of 0 or 1, where 0 means the image is the background, and 1 means the image is of an action. Then, a fault-tolerant mechanism is established to segment action suggestion intervals of different lengths, and the confidence of these images is taken as the score of the action suggestion interval. Finally, the intersection over the union of the action suggestion interval and the basic fact are calculated, and the non-maximum suppression algorithm is adopted. The location of the chopping board action instance is shown in Figure 4. The green box represents ground truths, the blue box represents a good prediction, and the red box represents a poor prediction.

**Figure 5:**
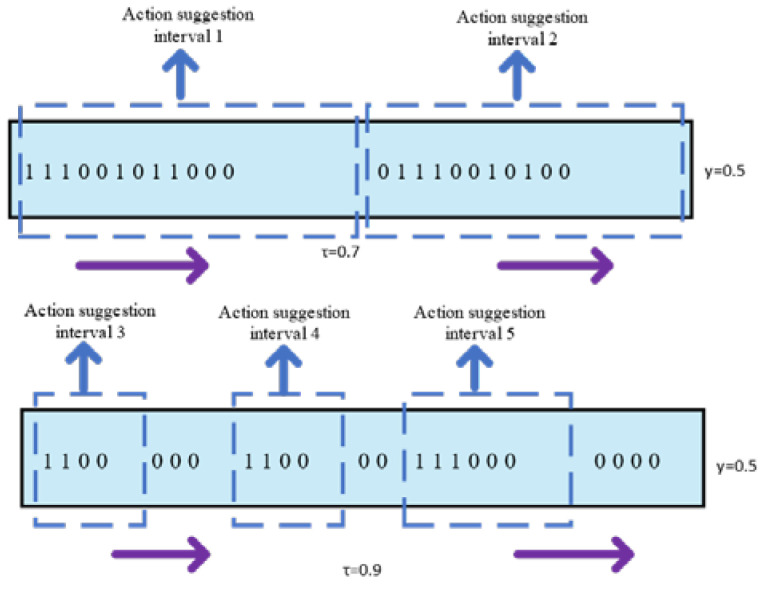
Schematic image of fault tolerance.

A robust fault-tolerant mechanism is designed by us, which is controlled by two design parameters: the score threshold *τ* and the tolerance threshold *γ*. If the confidence of the image is greater than *τ*, the image is marked as ‘1’, indicating that the image contains action. If the confidence of the image is less than *τ*, the image is marked as ‘0’, indicating this image is the background. For the fault tolerance threshold *γ*, we choose an image as a starting point, and recursively expand it by absorbing subsequent images, and terminate the expansion if the proportion of action suggestion interval label ‘1’ is less than *γ*. Obviously, this multi-threshold design enables us to remove background clips, generate action propose intervals of different lengths, and improve the accuracy of the video action location. As shown in Figure 5, the purple arrow indicates the extension direction of the action interval range.

Compared with images, a video not only needs to consider the spatial location information of the video frames but also needs to consider the temporal relationship between the video frames. Pretrained models on ImageNet [22] have achieved great success in the field of analog images, extending the 2D convolution in the picture to the 3D convolution in the video, and adding the idea of two streams to the 3D convolution network. [23] proposed two-stream inflated 3D convolutional neural network (Two-Stream I3D), where the spatial stream network extracts features of the objects from static RGB video frames and the temporal stream network extracts the motion information; finally, the outputs of these two layers are fused to obtain the action recognition results. A new dynamic sign language recognition network integrating dual stream 3D convolutional neural networks and attention mechanisms is proposed by [24]. The convolutional block attention module (CBAM) [25] is introduced into the I3D network to enable the network to learn the salient information in the image and make the important features in the image more prominent without affecting the performance of the original network. However, considering that the spatial location information and time-domain relationships are two different concepts, the learning methods of the two levels must be different for neural networks. Using the same network structure for spatial and temporal streams will lead to more redundant information in the stream information of the two layers in the final fusion stage, which wastes a large number of neuron parameters of the dual-stream convolutional network.

**Figure 6:**
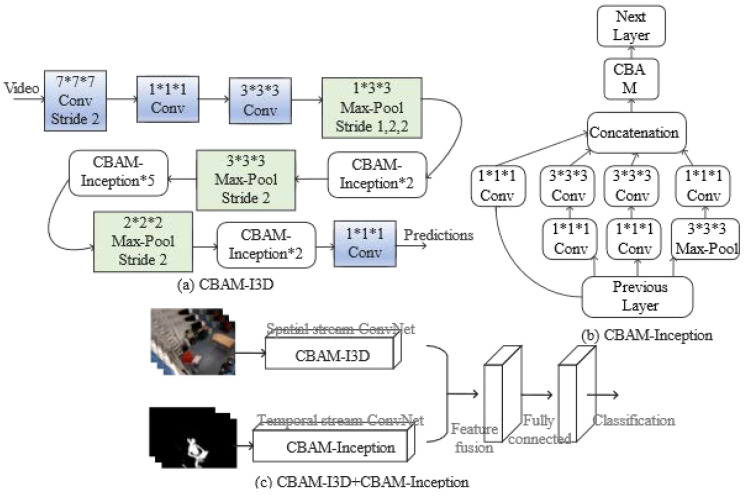
(**a**) CBAM-I3D network (**b**) CBAM-Inception network (**c**) Fusion two-stream heterogeneous 3D convolution and attention mechanism network.

Based on the above analysis, a network model integrating two-stream heterogeneous 3D convolution and attention mechanism is proposed, which introduces CBAM. The input is the original RGB image and the preprocessed optical flow map. The entire network model is shown in Figure 6. The spatial channel is added with CBAM after the Concatenation layer of the Inception module of I3D; the network connection method is shown in Figure 6a. The time channel only uses the Inception module of the I3D network, and also adds CBAM after the Concatenation layer. The network connection method is shown in Figure 6b. In addition to adding the attention mechanism CBAM, the spatial channel also improves the I3D network structure by: (1) Removing the first max pooling layer to prevent the loss of low-level features of the image; (2) Removing the final average pooling operation, so that the global information in the image can be preserved. Using the dual-stream heterogeneous network not only comprehensively considers the rich spatial feature information and motion features in the video frame, but also reduces the neural network parameters and improves the computational efficiency of the model.

The loss function used for network training is the cross-entropy function.

(6) $$loss=-\sum_{i}^{n}L(i)logS(i)$$

where *n* represents the total action label category, *L* represents the OneHot encoding of the true label, and *S* represents the probability of the predicted action label output by the Softmax layer. In the training process, the Backpropagation Algorithm is used to continuously update the network parameters to reduce the loss, and finally, the argmax function is used to obtain the category label of the action according to the maximum probability value.

### 3.2. Video Consistency Analysis Module

Video conformance checking includes predefined model and conformance checking. Predefined modules define standard Petri net process models; consistency is a calculation of the fit of process models extracted from noisy process logs and videos from predefined models.

#### 3.2.1. Predefined Model

**Figure 7:**
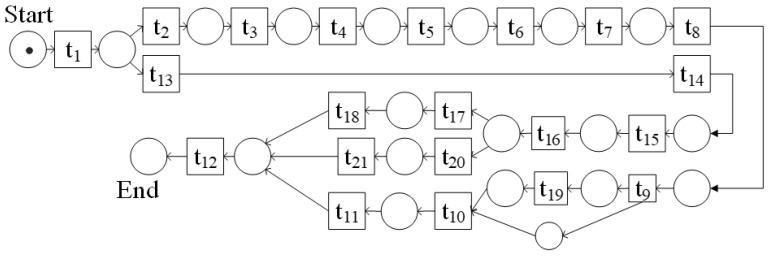
Example image of the predefined model part.

After analysis of the dataset, each process executor performs a series of actions in a natural state, and then a Petri net is used to represent a predefined model of the process, as shown in Figure 7. The specific descriptions of the events in Figure 7 are shown in Table 1. The following is a description of the predefined model where the process executor enters the scene and then has two options:

(1) Take the cutting board and rag, then put the cutting board and rag in order, then take the plate and cup, then put the plate and cup in order, then there are two options: ➀ Take a fork, knife, spoon, and put the fork, knife, spoon in that order; ➁ Take a fork, a spoon, and a knife, and then put the fork, spoon, and knife in that order.
(2) Take the cutting board, then put the cutting board, then take the rag, then put the rag, then take the plate, then put the plate, then take the spoon, then put the spoon, then open the cupboard, take the cup, put the cup, or just take the cup, put the cup.

No matter which utensils the executor sets first, the last process executor will leave the scene.

#### 3.2.2. Conformance Checking

In order to verify that the process model extracted from the video is more consistent with the actual execution of the business than the process model obtained from the process log containing noise, we did a comparative experiment to calculate the fitness of the two process models and the predefined model. Firstly, convert the process model extracted from the video, the process model obtained from the process log containing noise, and the predefined model into a directed graph, and then use the *GED_NAR* algorithm to calculate the fitness of the directed graph. Finally, the compliance results of the two process models and the predefined model are obtained.

**Figure 8:**
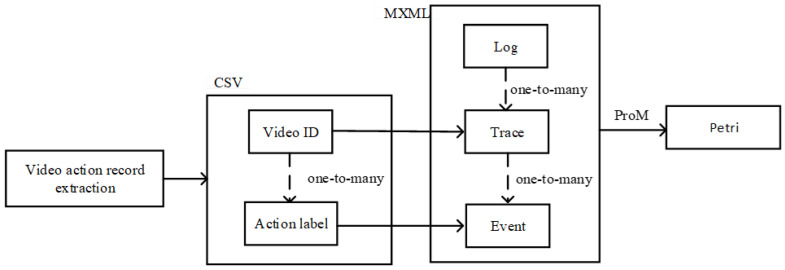
Flow chart of Petri net generation.

With action recognition, we can get the labels of each action category in the video, and after setting a unique ID for each video, we can build a CSV file containing two columns of attributes: “video ID” and “action label”. The “video ID” can represent the “case ID” in the process log, and the “action label” can represent the “activity” content in the process log. Finally, the video data process log file is obtained, and ProM is used to generate the Petri net model, as shown in Figure 8.

The relevant definitions of the *GED_NAR* method are given below:

**Definition** **1.** The fitness calculation of two directed graphs *G*1 = (*N*1*, E*1) and *G*2 = (*N*2*, E*2) is shown in Formula (7) where wskipn represents the cost coefficient of inserting and deleting action nodes, and wskipe represents the cost coefficient of inserting and deleting action relation edges; the value range is [0,1], which can be set according to actual needs with the default value set to 1.

(7) $$F_{GED}(G_{1},G_{2})=1.0-(wskipn\times fskipn+wskipe\times fskipe)/(wskipn+wskipe)$$

where *fskipn* represents the cost function of inserting and deleting action nodes, as shown in Formula (8), *skipn* represents the set of inserting and deleting action nodes, *N*_1_ and *N*_2_ represent the number of action nodes of the model.

(8) $$fskipn=(|skipn|)/(|N_{1}|+|N_{2}|)$$

where *fskipe* represents the cost function of inserting and deleting action relation edges, as shown in Formula (9), *skipe* represents the edge set of inserting and deleting, *E*_1_ and *E*_2_ represent the number of action relation edges of the model.

(9) $$fskipe=(|skipe|)/(|E_{1}|+|E_{2}|)$$

**Definition** **2.** If G = (*N*,*E*) is a directed graph, there are two nodes *p*, with *q* ∈ *N* satisfying *e = <p*, *q >* ∈ *E*, then < *p*, *q*> is called a node adjacency relationship, abbreviated as NAR, for a given directed graph. All node adjacencies constitute an adjacency set, denoted as NARs = {<*p*, *q* > |*e* = <*p*, *q*> ∈ *E*}.

The degree of fitness based on the adjacency relationship is calculated as shown in Formula (10):

(10) $$F_{NAR}(G_{1},G_{2})=(NARs1ꓵNARs2)/(NARs1ꓴNARs2)$$

**Definition** **3.** If *M*_1_ is a predefined model, *M*_2_ is an extracted process model, and *G*_1_ and *G*_2_ are directed graphs of *M*_1_ and *M*_2_, then the formula for the degree of fitness between the process model and the predefined model is shown in (11):

(11) $${Fitness}_{GED\_NAR}(M_{1},M_{2})=\alpha \times F_{GED}\left(G_{1},G_{2}\right)+\sigma \times F_{NAR}(G_{1},G_{2})$$

where *α* and *σ* are two coefficients, and the value range is [0,1], which can be set according to actual needs with the default value of 0.5 and *α* + *σ* = 1.

## 4. Experiments

Experiments analyzed the dataset, action localization, action recognition, and conformance checking.

### 4.1. Dataset

**Figure 9:**
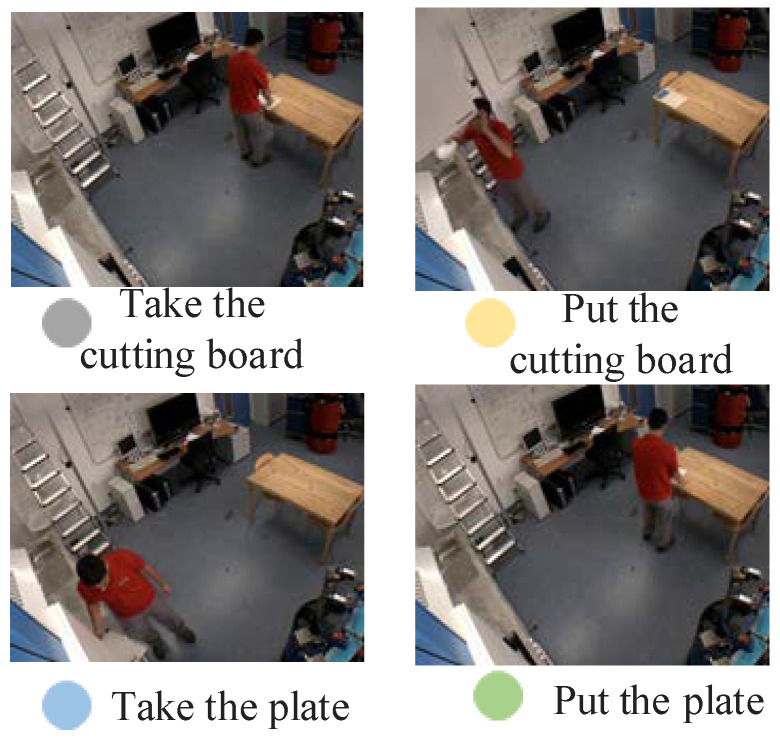
Sample Dataset.

At present, most of the datasets only involve action localization and action recognition, and there are very few datasets containing process information, so we use the public dataset TUM [26] for experiments, as shown in Figure 9. In the video, after the process executor enters the monitoring screen, he starts to take the cutting board and put it on the table. After the tableware is placed in an orderly manner, he finally leaves the monitoring scene. The duration of each video in the data set is in intervals of 1–2 min, and the action types in the video can be divided into taking a plate, placing a plate, etc. The “background class” is without any action. Finally, the video frame sequence is used to train and test the action localization and recognition networks.

### 4.2. Experiment of Action Localization

The action localization method is to design a two-class network to filter the video frames containing actions. The indicators used are Iou, Recall, Precision, and Aver-age-Precision. The final experimental results are shown in Table 2. We can find that the action localization scheme designed in this paper can locate the starting position and ending position of the action very well, which is mainly due to the fact that we not only set an action threshold to indicate how many points are counted as actions but also set a tolerance threshold to prevent the interference of noise, that is, the appearance of several video frames with background in consecutive frames still adds to the clip. Therefore, the action localization model designed in this paper has better results.

### 4.3. Experiment of Action Recognition

**Figure 10:**
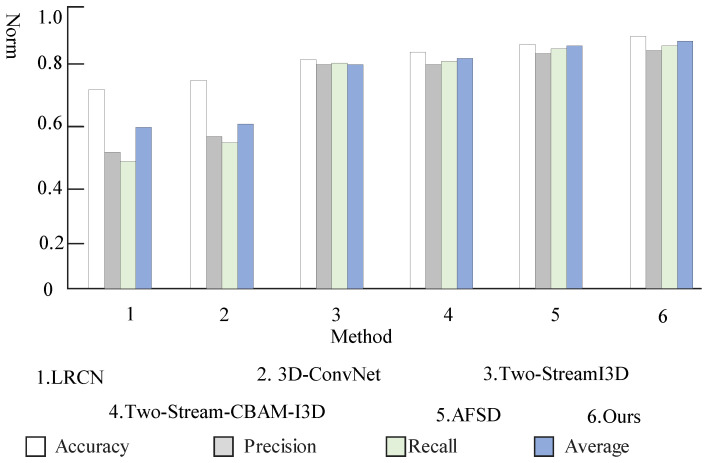
Performance comparison of action recognition.

We compare our model with other methods in Table 3, including LRCN [27], 3D-ConvNet [28], Two-StreamI3D [23], Two-Stream-CBAM-I3D [24], and AFSD [29]. The metrics used are accuracy, precision, recall, and a weighted composite evaluation index of the three for the experimental evaluation of action recognition. To avoid interference of other factors, the hyperparameter settings such as the learning rate, training rounds, and times used by the models are kept uniform. The results are shown in Table 3 and Figure 10. The various indicators of the fusion dual-stream heterogeneous 3D convolution and attention mechanism network model proposed in this paper are significantly improved compared to other models. Firstly, compared with the original Two-Stream I3D model, the accuracy rateincreased by 5%, the precision rate increased by 13.4%, the recall rate increased by 14.1%, and the average rate increased by 10.8%. Secondly, the two-stream heterogeneous structure reduces the complexity of the model and shortens the training time. Compared with the dual-stream CBAM-I3D model, the indicators are also slightly improved. Finally, the optical flow graphs generated by the preprocessing of the video data for the network inputs are improved to varying degrees. The analysis of the reasons for this shows that the network model proposed not only retains the originality of the input video information but also improves the expressive ability of the network, and automatically learns the importance of the spatial position information and temporal relationship of video frames, then according to the degree of importance, key features are enhanced and useless features are suppressed, so as to highlight the saliency information in the video frame. The introduction of the CBAM attention mechanism enables the network to learn more important spatial and temporal features in video frames without affecting the original network. The two-stream heterogeneous mode avoids repeated extraction of information, reduces the number of model calculation parameters and model size, and makes network convergence faster.

### 4.4. Conformance Checking Experimental Analysis

**Figure 11:**
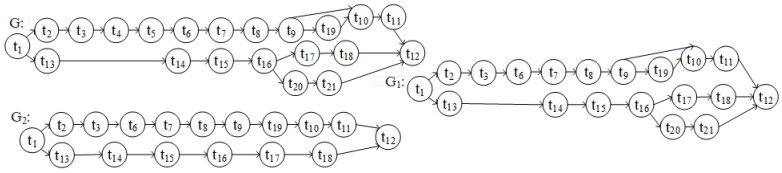
G: The directed graph corresponding to the predefined model, G_1_: The directed graph corresponding to the extracted process model, G_2_: The directed graph corresponding to the process model with noise.

Conformance checking analyzes how the process model matches the predefined model. In addition, to convert the process model into a directed graph with reference to the conversion rules proposed in [30] (as shown in Figure 11), the *GED_NAR* method is used to calculate the fitness of the model.

According to the Definition 1 of the *GED_NAR* method, the conversion of *G* to *G*_1_ requires deleting two nodes (*t*4, *t*5), deleting (*<t*3, *t*4*> <t*4, *t*5*> <t*5, *t*6*>*) three edges and adding (<*t*3, *t*6>) a side. Then the calculation process is as follows: |*skipn*| = 2, |*skipe*| = 4, *fskipn* = 2/(21 + 19) = 0.05, *fskipe* = 4/(23 + 21) = 0.09, F*_GED_* = 1.0 − (1 × 0.05 + 1 × 0.09)/(1 + 1) = 0.93.

Converting *G* to *G*_2_ requires deleting four nodes (*t*4, *t*5, *t*20, *t*21), deleting < *t*3, *t*4> <*t*4, *t*5> <*t*5, *t*6> <*t*9, *t*10> <*t*16, *t*20> <*t*20, *t*21> <*t*21, *t*12>) seven edges and adding (<*t*3, *t*6>) one edge. Then the calculation process is as follows: |*skipn*| = 4, |*skipe*| = 8, *fskipn* = 4/(21 + 17) = 0.11, *fskipe* = 8/(23 + 17) = 0.20, F*_GED_* = 1.0−(1 × 0.11 + 1 × 0.20)/(1 + 1) = 0.85.

According to the Definition 2 of the *GED_NAR* method, the node adjacency set of *G* is: NARs = {<*t*1, *t*2> <*t*2, *t*3> <*t*3, *t*4> <*t*4, *t*5> <*t*5*,t*6>…… <*t*16, *t*20> <*t*20, *t*21> <*t*21, *t*12>}. The node adjacency set of *G* is: NARs1 = {<*t*1, *t*2> <*t*2, *t*3> <*t*3, *t*6>……<*t*16, *t*20> <*t*20, *t*21> <*t*21, *t*12>}. The corresponding intersection relation is: NARsꓵNARs1 ={<*t*1, *t*2> <*t*2, *t*3>……<t16, *t*20> <*t*20*,t*21> <*t*21, *t*12>}; NARsUNARs1 = {<*t*1, *t*2> <*t*2, *t*3> <*t*3*,t*4> <t3, *t*6> <*t*4, *t*5>……<*t*16, *t*20> <*t*20*,t*21> <*t*21, *t*12>}. Therefore, the fitness degree of *G* and *G*_1_ obtained based on the node adjacency set is F*_NAR_*(*G*, *G*_1_) = 20/24 = 0.83.

According to the Definition 2 of the *GED_NAR* method, the node adjacency set of *G*2 is: NARs2 = {<*t*1, *t*2> <*t*2, *t*3> <*t*3, *t*6>……<*t*16, *t*17> <*t*17, *t*18> <*t*18, *t*12>}; The corresponding intersection relation is: NARsꓵNARs2 = {<*t*1, *t*2> <*t*2, *t*3>……<*t*16, *t*17> <*t*17, *t*18> <*t*18, *t*12>}; NARsꓴNARs2 = {<*t*1, *t*2> <*t*2, *t*3> <*t*3, *t*4> <*t*3, *t*6> <*t*4, *t*5> <t5, *t*6>……<*t*16, *t*20> <*t*20, *t*21> <*t*21, *t*12>}. Therefore, the fitness degree of *G* and *G*2 obtained based on the node adjacency set is: F*_NAR_*(*G*, *G*2) = 16/24 = 0.67.

According to the Definition 3 of the *GED_NAR* method, the process model extracted from the video and the predefined model can be obtained by using *GED_NAR* as:

Fitness*_GED-NAR_*(*G*, *G*_1_) = 0.5 × F*_GED_*(*G*, *G*_1_) + 0.5 × F*_NAR_*(*G*, *G*_1_) = 0.5 × 0.93 + 0.5 × 0.83 = 0.88.

According to the Definition 3 of the *GED_NAR* method, the process model obtained from the noisy process log and the predefined model can be obtained by using *GED_NAR* as:

Fitness*_GED-NAR_*(*G*, *G*_2_) = 0.5 × F*_GED_*(*G*, *G*_2_) + 0.5 × F*_NAR_*(*G*, *G*_2_) = 0.5 × 0.85 + 0.5 × 0.67 = 0.76.

From the experimental results, it can be seen that the fitness of the process model and the predefined model extracted from the video is greater than the fitness of the process model and the predefined model obtained from the process log containing noise. The experiment results show that the process model extracted from the video is more consistent with the actual execution process of the business.

## 5. Conclusions

Aiming at the problem that the process logs cannot be obtained effectively and the process logs generated by the information system will have noise, a method for extracting process models from videos and analyzing their consistency is proposed by us. Firstly, the video data preprocessing removes the background information irrelevant to the moving target in the video picture and only retains the spatiotemporal interest point area. Secondly, a binary classification network is used to generate action suggestion intervals of different lengths, which are then inputed to the fusion dual-stream heterogeneous, the three-dimensional convolution and attention mechanism network to classify actions. Finally, consistency detection is carried out with the predefined model and with the process model obtained from the process logs containing noise and the process model extracted from the video.

The method proposed in this paper is only for single-person video process modeling, and cannot handle multi-person collaborative business processes well. The next step is to study how to model a multi-person collaborative video process and conduct conformance checking.

## Figures and Tables

**Table 1:** Correspondence table of event number and name.

Event Sequence	Event Name	Event Sequence	Event Name
t_1_	Enter the scene	t_2_	Take the cutting board
t_3_	Put the cutting board	t_4_	Take the rag
t_5_	Put the rag	t_6_	Take the plate
t_7_	Put the plate	t_8_	Take the spoon
t_9_	Put the spoon	t_10_	Take the cup
t_11_	Put the cup	t_12_	Leave the scene
t_13_	Take the cutting board and the rag	t_14_	Put the cutting board and the rag
t_15_	Take the plate and the cup	t_16_	Put the plate and the cup
t_17_	Take the fork, knife, and spoon	t_18_	Put the Fork, knife, and spoon
t_19_	Open cupboard	t_20_	Take the fork, spoon, and knife
t_21_	Put the fork, spoon, and knife		

**Table 2:** Action positioning indicator (%).

Iou			0.3				0.5				0.7	
Recall	35.6%	37.0%	24.1%	1	18.5%	17.4%	10.3%	1	8.4%	9.0%	10.3%	1
Precision	94.1%	71.2%	50.0%	14.3%	71.4%	57.1%	21.4%	14.2%	30.1%	35.7%	21.4%	14.2%
AP			38.9%				23.9%				16.3%	

**Table 3:** Performance evaluation table for action recognition (%).

Network	Accuracy	Precision	Recall	Average
LRCN	71.3	54.5	52.8	59.5
3D-ConvNet	76.2	58.2	57.0	63.8
Two-StreamI3D	82.0	79.3	80.4	80.5
Two-Stream-CBAM-I3D	85.2	79.3	80.2	81.5
AFSD	86.1	80.5	81.2	82.6
Ours	87.0	83.6	85.4	85.3

## Data Availability

Enquiries about data availability should be directed to the authors.
